# Fetal cell microchimerism and susceptibility to COVID-19 disease in women

**DOI:** 10.1007/s15010-023-02006-x

**Published:** 2023-03-01

**Authors:** Valentina Cirello, Marina Lugaresi, Alessandro Manzo, Eva Balla, Gerardina Fratianni, Francesca Solari, Luca Persani, Laura Fugazzola, Irene Campi

**Affiliations:** 1 Department of Endocrine and Metabolic Diseases, Istituto Auxologico Italiano IRCCS, Milan, Italy; 2 Department of Pathophysiology and Transplantation, University of Milan, Milan, Italy; 3 Department of Biotechnology and Translational Medicine, University of Milan, Milan, Italy; 4 Department of Cardiovascular Neural and Metabolic Sciences, Istituto Auxologico Italiano, IRCCS, Milan, Italy; 5 Department of Occupational Medicine Unit, Istituto Auxologico Italiano IRCCS, Milan, Italy

**Keywords:** COVID-19, SARS-CoV-2, Pregnancy, Fetal cell microchimerism, Microchimeric cells

## Abstract

**Purpose:**

The clinical outcome of COVID-19 disease is worse in males, and the reasons of this gender disparity are currently unclear, though evidences point to a combination of biological and gender-specific factors. A phenomenon unique to the female gender is the fetal cell microchimerism (FCM), defined as the presence of fetal microchimeric cells in maternal organs and in the circulation for years after delivery and usually evaluated by assessing the presence of male cells or DNA in a woman. In the present case–control study, we aimed to evaluate the possible effect of pregnancy and related FCM on the susceptibility to SARS-CoV-2 infection and on the clinical course and outcome of COVID-19.

**Methods:**

One hundred twenty-three women with a previous male pregnancy, comprising 63 COVID-19 cases and 60 healthy controls were enrolled. The presence of blood male DNA was assessed by the amplification of the Y-chromosome specific gene SRY.

**Results:**

The prevalence of male DNA of presumed fetal origin was significantly higher in healthy controls than in COVID-19 cases (70 vs 44.4%, *P* = 0.0044; OR 0.3429, 95% CI 0.1631–0.7207, *P* = 0.0047). Among women affected with COVID-19, the presence of male FCM did not significantly influence the severity of the disease, though the 8 deceased women studied were all FCM negative.

**Conclusion:**

This is the first case–control study reporting the prevalence of FCM in COVID-19 and healthy women. Overall, our data seem to suggest a role for FCM in the protection towards the SARS-CoV-2 infection with a possible positive impact on clinical outcome.

**Supplementary Information:**

The online version contains supplementary material available at 10.1007/s15010-023-02006-x.

## Introduction

SARS-CoV-2 virus causes a severe respiratory disease, defined coronavirus disease 2019 (COVID-19). Deaths result from acute respiratory distress syndrome (ARDS), acute respiratory failure, coagulopathy, septic shock, and metabolic acidosis [1]. Cardiovascular complications comprise arrhythmias, acute cardiac injury, and shock. Case fatality is higher in patients over 80 years and in individuals with comorbidities [1]. Early studies in China and Italy indicated a higher susceptibility of males to COVID-19 infection [2–5], but further epidemiological evidences showed that the susceptibility to SARS-CoV-2 infection is equally likely between males and females [6–8]. Nevertheless, in males the clinical outcome is worst and the prevalence of fatal cases is higher. On the other hand, being a pre-menopausal woman seems to be a protective factor for COVID-19 severity and fatality [9–11]. The mechanism accounting for the reduced case fatality rate in women are currently unclear, but warrants attention for the possible impact on the management and treatment of these patients. Evidences suggest that sex-based differences in the expression of the angiotensin-converting enzyme 2 (ACE2) receptor and the transmembrane protease serine 2 (TMPRSS2), which are responsible for the successful entry of SARS-CoV-2 in the body, may explain this disparity [9]. Moreover, sex-based difference in immunological responses and in behaviors (smoking and prevalence to comorbidities) may influence clinical outcome [12–14].

Interestingly, a unique phenomenon has been described occurring only in females and named fetal cell microchimerism (FCM), which consists in the acquisition of fetal microchimeric cells during pregnancy, and in their persistence in maternal organs and in the circulation for decades after delivery [15–18]. The presence of two genetically distinct population of cells in an individual as a consequence of the transfer of a low number of cells from one individual to another can occur in cases of blood transfusion, organ transplantation and more frequently during pregnancy. In the latter, a bidirectional trafficking of maternal and fetal cells can be observed starting from 4 to 6 weeks of gestation and can persist in blood and tissues for decades [18]. We previously observed a protective role of FCM toward both the onset and the progression of thyroid cancer and autoimmune thyroid disorders [19–22]. We demonstrated the vascular and tissue localization of microchimeric cells, highlighting the ability of those cells to migrate to damaged tissues [20, 22]. Importantly, it has been reported that FCM could be involved in the control of viral infections, [23] but no data are yet available regarding a possible role in modulating the clinical expression of SARS-CoV-2 infection in female patients. In the present case–control study, we aimed to evaluate the possible effect of pregnancy and related FCM on the susceptibility to SARS-CoV-2 infection and on the clinical course and outcome of COVID-19. Since the most widely used procedure for evaluating FCM is assessing, by a highly sensitive PCR, the presence of male DNA in a woman, we enrolled female patients with a previous male pregnancy.

## Patients and methods

### Patients and healthy controls enrollment

**Fig. 1:**
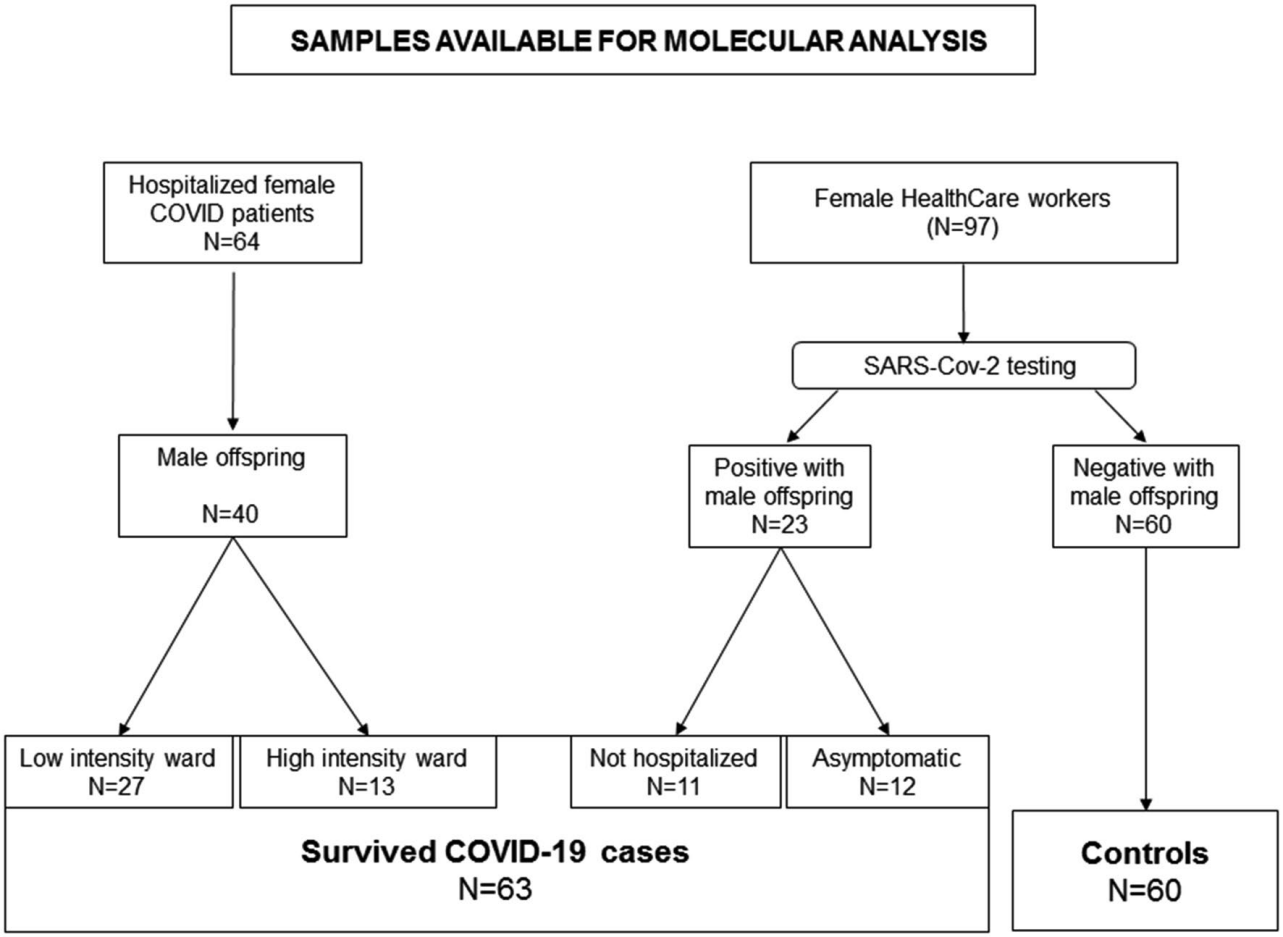
Enrollment flow diagram of COVID-19 cases and Controls included in the study

Between March 2020 and December 2020, we studied 161 COVID-19 female patients, of which 64 consecutive hospitalized patients and 97 HealthCare workers with a positive SARS-CoV-2 test. The study period was limited to December 2020 before the development and the availability of SARS-Co-V-2 vaccine, as we were interested in the natural history of the disease, that was strikingly modified by the mass vaccination campaign. After the exclusion of cases who had no pregnancy or female offspring or insufficient data, we analyzed 63 women (COVID-19 cases) with a previous male pregnancy and affected with SARS-CoV-2 infections of variable severity (Fig. 1). Among these women: 40 had a mild to severe respiratory insufficiency hospitalized in the COVID Units at our Institution, 11 were treated at home, and 12 were asymptomatic HealthCare workers diagnosed by a positive naso-pharyngeal swab (NPS) (*n* = 5) or by serological screening (*n* = 7). Blood samples were taken at the first follow-up visit one to three months after discharge. We also enrolled 60 unaffected female controls (Controls) with male offspring, among HealthCare workers (Fig. 1). They were selected among the 304 female workers screened for COVID-19 at our Institution, according with the indications of the Italian Ministry of Health, with periodical nasal swab every 15 or 30 days starting from April 2020. In addition, they were submitted to a serological screening every 3 months until the end of 2021. Noteworthy, none of the controls selected for this study had a positive test or reported fever or other symptoms suggestive for airways infections during the enrollment period and in the subsequent follow-up (January 2021-Nov 2021). The study was approved by the ethics committee of Istituto Auxologico Italiano (COV-endo study, n 05C021). The participants or their parents gave informed consent to include their clinical data in the present study.

### Molecular and serological analyses

SARS-Cov-2 infection was confirmed in all symptomatic patients by RT-PCR from NPS. For the serological screening we measured both anti-SARS-CoV-2-RDB-Spike (Elecsys Anti-SARS-CoV-2 S, Roche Diagnostics GmbH, Mannheim, Germany) and anti-SARS-CoV-2-N protein total Ig (Elecsys Anti-SARS-CoV-2, Roche Diagnostics GmbH, Mannheim, Germany), in order to discriminate between natural infection and immunization by vaccine, which was at that time administered to some Health System operators.

### DNA extraction and PCR amplification of SRY gene

Genomic DNA was extracted from whole blood samples using the DNA Extraction Kit BACC2 (GE Healthcare, Buckinghamshire, UK). For the detection of the human Y chromosome, DNA was submitted to two rounds of PCR (35 cycles each) using primers specific for the SRY locus and corresponding to the region upstream of the SRY-coding region, as previously described [19]. We previously demonstrated that this method has a sensitivity of 1 male cell/1 million female cells. Each PCR analysis was repeated twice, two negative controls (DNA from two pre-pubertal girls) and two positive controls (DNA from two men) were included, and special care was taken to avoid external contamination (Supplementary Fig. 1). In particular, all of the samples were handled by a female technician, positive displacement micropipettes were used, and DNA extraction, PCR preparation, and analyses were conducted in separate rooms. Samples were electrophoresed in 2% agarose gels and visualized and photographed on an U.V. trans-illuminator (BioDoc-It Imaging System, UVP, Cambridge, UK).

### Statistical analysis

Possible differences in the clinical features between COVID-19 patients and healthy controls were assessed by *t*-test and *Chi*-square analysis, as appropriate. Odds ratio (OR) and 95% confidence intervals (CI) were used to assess the strength of the association between the presence/absence of FCM and the presence/absence of the COVID-19 disease. Possible differences in associated comorbidities, in the clinical course and symptoms were assessed in COVID-19 patients by *t test* and *Chi-*square analysis, as appropriate. The difference between values was considered significant when *p* < 0.05. A multivariate logistic regression model was employed to determine odds ratios (OR) for hospitalization in patients affected with COVID-19. The predictive model was determined by stepwise selection using a *P* value = 0.05 as the entry value for the model. All tests were performed using the Version 20.014 of the Med-Calc Software (B-8400 Ostend, Belgium).

## Results

### Clinical features and peripheral blood FCM in parous women with COVID-19 or healthy

**Fig. 2:**
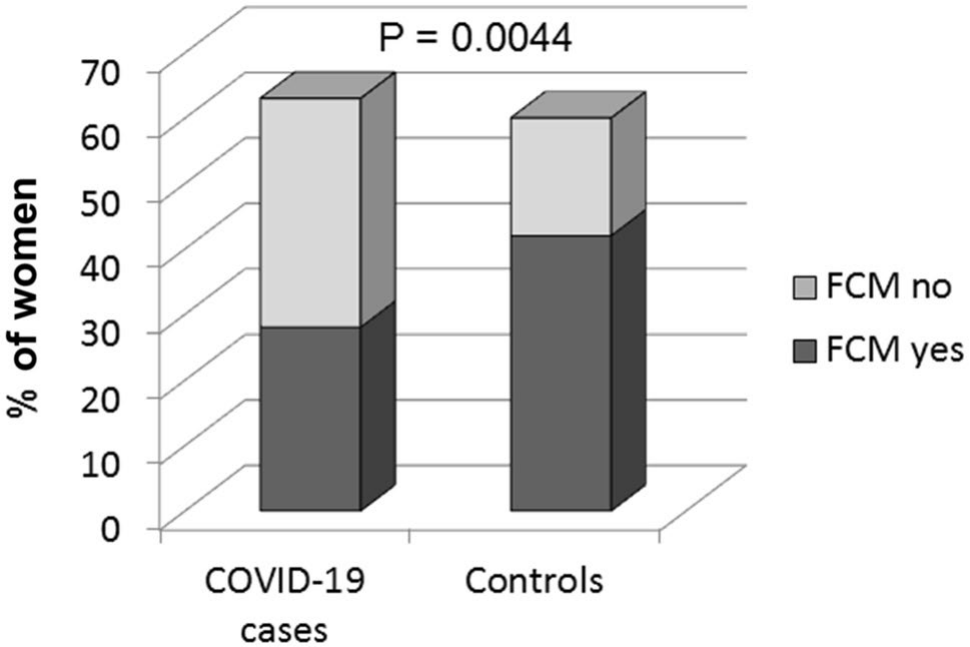
Prevalence of fetal cell microchimerism (FCM) in women with COVID-19 and Controls

COVID-19 cases and Controls did not significantly differ with respect to the age at enrollment (mean age 57.54 and 54.1 years, respectively) and several clinical features related to pregnancy history (age at the last male pregnancy, number of pregnancies, presence and number of miscarriages, number of male children, time elapsed from the last male pregnancy to sampling, in vitro fertilization, cesarean section) (Table 1). No differences were observed also for other clinical parameters, which could be potential sources of different forms of chimerism, such as maternal miscarriages, the presence of twin brothers, blood transfusion and organ transplant (Table 1). Interestingly, the presence of male DNA of presumed fetal origin had a significantly higher prevalence in controls than in COVID-19 cases (70 vs 44.4%, *P* = 0.0044 by Chi-square, OR 0.3429, 95% CI 0.1631–0.7207, *P* = 0.0047 by logistic regression) (Fig. 2). Within both groups, women positive for the presence of FCM were not significantly different from those without FCM considering the age at enrollment and several clinical parameters related to pregnancy history and other sources of chimerism (Table 2).

**Table 1:** Clinical features of parous COVID-19 patients (*n* = 63) and controls (*n* = 60)

	Clinical features	COVID-19 cases	Controls	*p*
Pregnancies history	Age at enrollment, years (range)	57.5 (24–83)	54.1 (38–83)	0.060
	Age at last male pregnancies, years (range)	32 (15–47)	33 (21–55)	0.545
	N of pregnancies (range)	2.6 (1–7)	2.2 (1–4)	0.123
	N of miscarriages (range)	0.5 (0–4)	0.3 (0–2)	0.627
	N of male children (range)	1.5 (1–5)	1.3 (1–3)	0.053
	Time elapsed from the last male pregnancy to sampling, years (range)	25 (1–56)	21 (1–53)	0.115
	Miscarriages (yes/no)	21/40	18/38	0.794
	IVF (yes/no)	2/29	5/44	0.565
	Cesarean section (yes/no)	6/25	15/31	0.203
Other causes of chimerism	Maternal miscarriages (yes/no)	3/27	7/45	0.647
	Twin Brothers (yes/no)	1/27	5/43	0.289
	Blood transfusion (yes/no)	2/29	6/46	0.450
	Organ transplant (yes/no)	0/31	1/50	0.748

*IVF* in vitro fertilization

**Table 2:** Clinical features of parous COVID-19 patients and Controls with or without fetal cell microchimerism (FCM)

Clinical features	COVID-19 Cases *N* = 63			Controls *N* = 60		
	FCM-ve *N* = 35	FCM + ve *N* = 28	*p* values	FCM-ve *N* = 18	FCM + ve *N* = 42	*p* values
Age at enrollment (years)	60.5	53.9	0.052	57.3	52.7	0.287
Age at last male pregnancies (years)	32.5	31.3	0.450	30.9	33.8	0.143
N of pregnancies	2.4	2.9	0.158	2.188	2.214	0.892
N of pregnancies (= 1/ > 1)	26/8	25/3	0.192	6/10	10/32	0.301
N of miscarriages	0.3636	0.571	0.2682	0.438	0.3	0.510
N of male children	1.382	1.679	0.189	1.382	1.679	0.900
Time elapsed from the last male pregnancy to sampling (years)	27.7	21.6	0.072	26.2	18.7	0.165
Miscarriages (yes/no)	9/24	12/16	0.206	6/10	12/28	0.591
IVF (yes/no)	1/17	1/12	0.814	2/12	3/32	0.555
Cesarean section (yes/no)	5/13	1/12	0.169	5/9	10/22	0.769
Maternal miscarriages (yes/no)	1/17	2/10	0.329	3/13	4/32	0.544
Twin brothers (yes/no)	1/17	0/10	0.456	0/14	5/29	0.134
Blood transfusion (yes/no)	1/17	1/12	0.814	2/14	4/32	0.886
Organ transplant (yes/no)	0/18	0/13	–	1/15	0/35	0.139

IVF in vitro fertilization, − ve negative, + ve positive

### Clinical features of parous women affected with COVID-19 resulted positive or negative for circulating FCM

Among women affected with COVID-19, the presence of male FCM did not influence the severity of the disease, regarding symptoms, clinical course and associated comorbidities (Table 3). Concerning the clinical course, women with or without FMC had comparable length of the diseases (assessed as the duration of acute symptoms), and comparable length of the viral clearance (measured as the time elapsed between the first positive and the first negative naso-pharyngeal swab-NPS). The type of ventilation for hospitalized case (none/low–high flow oxygen/pressure support) was not statistically different between FMC positive and negative women, too. The number of patients with known risk factor for severe COVID-19 infections (hypertension, chronic obstructive pulmonary disease-COPD, cardiovascular diseases-CVD, obesity, thyroid diseases, epilepsy, age older than 65 years) was not different between FMC positive and negative women. Finally, the risk of being hospitalized was not influenced by FMC, but was increased by the age older than 65 years (OR 38.64, RR 2.05, *P* < 0,0001), the presence of comorbidities such as hypertension (OR 11.61, RR 1.92, *P* = 0.0002), obesity and overweight (OR 4.02, RR 1.63, *P* = 0.016) (*data not shown*). Nevertheless, we had the opportunity to test the blood samples of 8 parous women deceased as a consequence of SARS-CoV-2 infection, and they were all FCM negative.

**Table 3:** Clinical features of 63 parous COVID-19 women according to the presence or the absence of fetal cell microchimerism (FCM)

	Clinical features of COVID-19 patients	FCM-ve (*n* = 35)	FCM + ve (*n* = 28)	*p *values
Associated comorbidities	Older age (≤ 65/ > 65 years old)	11/24	7/21	0.578
	Hypertension (yes/no)	12/23	11/17	0.685
	CVD (yes/no)	4/31	2/26	0.568
	COPD (yes/no)	2/33	0/22	0.202
	Obesity/Overweight (yes/no)	16/19	8/16	0.346
	Thyroid diseases (yes/no)	9/26	4/24	0.269
	Epilepsy (yes/no)	0/35	1/27	0.264
Clinical course	Disease’s duration (days ± SD)	29.63 ± 27.18	25.27 ± 15.4	0.959
	NPS positivity (days ± SD)	20.11 ± 11.54	19.80 ± 13.58	0.755
	Symptomatic /asymptomatic	28 (80)	22 (78.6)	0.646
	Hospitalization (yes/no)	23 (65.6)	17 (60.7)	0.685
	No ventilation/ low and high-flow oxygen/pressure support	7/8/8	8/4/5	0.543
	Home care/sub-intensive care/intensive care	12/15/8	11/12/5	0.863
Symptoms	Asthenia	8 (22.9)	8 (28.6)	0.491
	Anosmia (yes/no)	6/29	4/22	0.856
	Headache (yes/no)	3/32	3/23	0.703
	Dysgeusia/Ageusia (yes/no)	8/27	5/21	0.734
	Dyspnea (yes/no)	15/20	11/15	0.966
	Chest pain (yes/no)	2/33	3/23	0.416
	Fever (yes/no)	24/11	16/10	0.571
	Gastroenteritis (yes/no)	4/31	3/23	0.990
	Myalgia/Arthralgia (yes/no)	3/32	5/21	0.226
	Upper respiratory tract symptoms (yes/no)	9/26	6/20	0.815
	Tachycardia (yes/no)	0/35	2/24	0.100
	Cough (yes/no)	13/22	9/17	0.840

*CVD* cardiovascular disease, *COPD* chronic obstructive pulmonary disease, *NPS* naso-pharingeal swab, − ve negative, + ve positive

### Possible impact of pregnancy on COVID-19 incidence

In order to evaluate the possible impact of pregnancy (regardless of the microchimeric status) on COVID-19 incidence in premenopausal women, we evaluated the pregnancies history of 304 female HealthCare workers among our Institutional Preventive Medicine Cohort. Among these women, 84 were cases with a positive SARS-CoV-2 test and 220 were never affected with COVID-19. No difference was observed in the number of nulliparous women or women with offspring between the two groups (Chi-square *P* = 0.557, Supplementary Table 1).

## Discussion

We report data on the first study investigating the possible role of peripheral blood fetal cell microchimerism (FCM) in COVID-19 disease. In parous women, FCM was significantly more prevalent in the blood of healthy women than in those with COVID-19 disease, suggesting a protective role for FCM against the SARS-CoV-2 infection in women. On the other hand, the outcome of the disease was not different among microchimeric positive or negative COVID-19 women, though 8 deceased women were FCM negative. This is probably due to the fact that most cases survived to COVID-19, while differences in the outcome could emerge by the analysis of a higher number of deceased women. We previously demonstrated a protective role for circulating FCM toward the onset and the progression of thyroid cancer [19–22] and the development of autoimmune disease [20]. Interestingly, a positive role of fetal microchimeric cells was also reported for viral infections, such as that from the hepatitis C virus. In particular, male fetal cells were demonstrated indistinguishable from the female cells in the liver specimens, suggesting that fetal stem cells acquired after pregnancy may have differentiated into hepatic cells acting as an alternate source of tissue repair [23].

Despite SARS-CoV-2 infection is equally prevalent in males and females, the clinical outcome is worse in males [9–11, 13, 24]. The mechanism accounting for the reduced case fatality rate in women are currently unclear, but evidences suggest that the sex disparity of COVID-19-related morbidity and mortality could be explained by a combination of biological sex differences (differences in chromosomes, reproductive organs, and related sex steroids) and gender-specific factors (differential behaviors and activities by social and cultural/traditional roles) [9, 11, 13]. Moreover, it is well-known that women show more robust immune responses following infection from a variety of pathogens (i.e. hepatitis A and B, Epstein-Barr virus, and West Nile virus), leading to decreased mortality [14, 25].

In order to evaluate if the observed result could be due to some factors related to pregnancy, we compared COVID-19 cases and controls, and found no differences in the prevalence of pregnancies, indicating that the protection against the infection is not related to pregnancy, but exclusively to FCM, at least in the present series. Interestingly, microchimeric cells have been reported in tonsils and adenoids, the organs of first line defense against infective agents of the upper airways [26]. Moreover, microchimeric cells are particularly abundant in lungs, in both humans and mice, as they contains the first capillary bed through which blood from the placenta passes [27–30].

Thus, we are tempting to speculate that active fetal microchimeric immune cells located in the upper airways and in the lungs could act as a barrier towards SARS-CoV-2 infection, strengthening the action of maternal immune system. It is also possible that fetal microchimeric cells, for which a multilineage potential plasticity have been demonstrated [31], could differentiate in respiratory epithelium cells in order to replace and repair damaged lung tissue. Our hypothesis is supported by the observation that fetal cells, especially within the lung of pregnant female mice, express a variety of surface markers typical of both immature and mature cell types [32]. These cells preferentially traffic to the maternal lung, where they may play an important role in recovery or protection from lung disease. More mature cells would be more likely to initiate a host immune response, while immature cells would be more likely to contribute to tissue repair [33]. Their presence in the lungs may be one explanation for the sex differences observed in the prevalence and prognosis of some lung diseases, COVID-19 among them.

A limitation of our study is the low number of samples from deceased women to reliably demonstrate a positive effect of FCM on the final outcome of the infection, too.

It is worth to emphasize that we focused on the period before the mass vaccination campaign, since vaccines influence the natural history of communicable diseases, by reducing the susceptibility to infections and viral circulation. Therefore, the study of any natural factor modulating host immune responses, including FMC, would have been masked by the stronger effects of the vaccine.

## Conclusions

This is the first case–control study in which the prevalence of FCM has been reported in both COVID-19 and healthy women with a previous male pregnancy. Overall, our data seem to suggest a role for FCM in the control of SARS-CoV-2 infection with a positive impact on clinical outcome. Studies are currently in progress to analyze if male fetal cells can be documented in the COVID-19 affected tissues, eg. lung, with a possible repair function as previously observed in thyroid cancer [22] and in hepatitis C [23].

## Supplementary Information

Below is the link to the electronic supplementary material.

Supplementary file1 (DOCX 15 KB)

Supplementary file2 Representative image of SRY fragment amplification of 5 healthy controls and 5 COVID-19 cases performed by 2-rounds PCR and run on 2% agarose gel. Two negative controls (DNA from two prepubertal girls), two positive controls (DNA from two men), the re-amplified blank from the first PCR (Blank 1) and that from the second reaction (Blank 2) were included. MW, molecular weight (TIF 154 KB)

## Data Availability

Data will be available upon request.
