# Supplementary material for: Fetal cell microchimerism and susceptibility to COVID-19 disease in women
Source: Infection. 2023 Mar 1;51(4):1071–8. doi: 10.1007/s15010-023-02006-x (PMC9975871; doi:10.1007/s15010-023-02006-x)
Supplement: Supplementary file 1 — Supplementary file1 (DOCX 15 KB) [file 15010_2023_2006_MOESM1_ESM.docx]

**Supplementary Table I:** Impact of pregnancy on COVID-19 incidence in premenopausal female healthcare workers of Istituto Auxologico Italiano.

|  | **PREVENTIVE MEDICINE COHORT** | | |
| --- | --- | --- | --- |
|  | **COVID-19 cases**  **N=84 (%)** | **Controls**  **N=220 (%)** | ***p values*** |
| **Nulliparous** | 26 (30.9) | 78 (35.5) | 0.557 |
| **Male offspring/female/unknown gender** | 23/3/32 (69.1) | 60/7/75 (64.5) |  |
