# Supplementary figures and images for: Fetal cell microchimerism and susceptibility to COVID-19 disease in women
Source: Infection. 2023 Mar 1;51(4):1071–8. doi: 10.1007/s15010-023-02006-x (PMC9975871; doi:10.1007/s15010-023-02006-x)

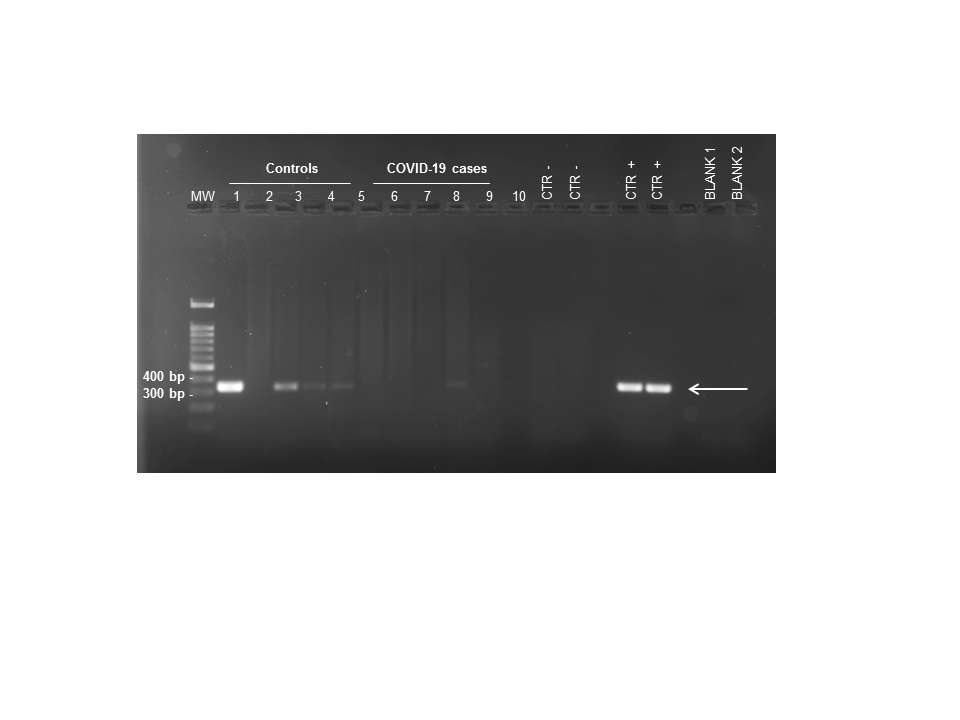

Supplement: Supplementary file 2 — Supplementary file2 Representative image of SRY fragment amplification of 5 healthy controls and 5 COVID-19 cases performed by 2-rounds PCR and run on 2% agarose gel. Two negative controls (DNA from two prepubertal girls), two positive controls (DNA from two men), the re-amplified blank from the first PCR (Blank 1) and that from the second reaction (Blank 2) were included. MW, molecular weight (TIF 154 KB) [file 15010_2023_2006_MOESM2_ESM.tif]
